# Exercise Training but not Curcumin Supplementation Decreases Immune Cell Infiltration in the Pancreatic Islets of a Genetically Susceptible Model of Type 1 Diabetes

**DOI:** 10.1186/s40798-017-0082-3

**Published:** 2017-04-04

**Authors:** Leandro Kansuke Oharomari, Camila de Moraes, Anderson Marliere Navarro

**Affiliations:** 1 Department of Food and Nutrition, Pharmaceutical Sciences College, Araraquara, São Paulo Brazil; 2 School of Physical Education and Sport of Ribeirão Preto, University of São Paulo, Ribeirão Preto, São Paulo Brazil

**Keywords:** Autoimmunity, Type 1 diabetes, Insulitis, Exercise training, Bioactive compound, Curcumin

## Abstract

**Background:**

The main mechanism involved in the pathogenesis of autoimmunity is an uncontrolled inflammatory response against self-antigens. Therefore, anti-inflammatory factors, such as the intake of bioactive compounds and a physically active lifestyle, may decrease or cease the development of autoimmune diseases. Type 1 diabetes (T1D) is an autoimmune disease characterized by pancreatic β cell destruction. The non-obese diabetic (NOD) mouse is a model of spontaneous T1D and is the model most similar to human disease.

**Methods:**

To determine the effects of exercise training and curcumin supplementation on T1D progression, 48 NOD mice, 5 weeks old, were randomly divided into four groups: control, curcumin supplementation, trained, and trained plus curcumin. Every 2 weeks, blood glucose was measured using a glucometer. At the end of 20 weeks, a histopathological procedure was used to assess immune cells infiltration into pancreatic β cells (insulitis).

**Results:**

Moderate intensity exercise training has the potential to protect pancreatic β cells against an immune response in vivo. However, curcumin supplementation failed to attenuate insulitis in NOD mice.

**Conclusions:**

These data provide evidence that exercise training can mitigate T1D development in genetically susceptible mice.

## Key Points

- Exercise training decreased immune cells infiltration in vivo in a spontaneous mouse model of type 1 diabetes.
- Curcumin supplementation did not attenuate insulitis alone or improve exercise training effects.
- The effect of exercise training is present regardless of IL-6 and TNF-alpha alteration.

## Background

Intense inflammatory response is a main feature of autoimmune diseases. Type 1 diabetes (T1D), for instance, occurs due to a chronic inflammatory response with enough severity to destroy most of the pancreatic β cells [1]. The incidence of some autoimmune diseases, including T1D, has been increasing worldwide over the past decades [2–5], and environmental factors are the most accepted explanation for this phenomenon. For example, vitamin D status [6] and the hygiene hypothesis [7] are being investigated. Currently, physical inactivity and processed food intake during childhood appear to be linked to the development of chronic [8–10] and inflammatory disease [11] in early stages of life. Furthermore, the Overload Hypothesis suggests an association between a modern lifestyle and beta cell overload, which could make these cells more vulnerable to an autoimmune response [12].

Intervention using functional foods with anti-inflammatory properties could decrease or halt the autoimmune response, leading to a delay or even prevention of autoimmunity. Dietary patterns characterized by high bioactive compounds, such as a Mediterranean diet, promote an “anti-inflammatory environment” in humans [13]. Among several compounds, curcumin, a polyphenol found in the rhizome of *Curcuma longa*, is one of the most studied bioactive compounds due to its anti-inflammatory properties [14–16]. Additionally, studies have shown that physical exercise also promotes anti-inflammatory responses through several mechanisms, such as increasing antioxidant activity [17], releasing myokines and hormones [18], modulating immune cell metabolism [19], and decreasing inflammation signaling [20].

Currently, the non-obese diabetic (NOD) mouse, a model of spontaneous T1D, is the model that is most similar to human T1D [21]. Over the last 30 years, it has been used in research to improve the science of autoimmunity [22]. As in humans, T1D occurs in NOD mice due to immune cell infiltration into pancreatic islets (insulitis) and subsequent destruction of β cells. Additionally, it has been demonstrated by ample literature that TNF-α, a pro-inflammatory cytokine, has an important role in the pathogenesis of T1D in NOD mice [23–27].

Some studies have tested bioactive compounds in NOD mice. Supplementation with cocoa flavonoids or green tea catechin reduced diabetes incidence in this animal model [28, 29]. However, no studies have tested the potential of exercise training to prevent T1D in NOD mice. Therefore, this study aimed to investigate the effects of exercise training and curcumin supplementation on T1D progression in NOD mice.

## Methods

### Animals

Forty-eight female NOD mice, 5 weeks old, were randomly divided into four groups (*n* = 12): control (C), curcumin supplementation (CUR), trained (T), and trained + curcumin supplementation (TC). The experimental protocol lasted 20 weeks. Animals were housed collectively (four animals per cage) and kept in conventional conditions (22–24 °C and 12-h light-dark cycle) with unlimited access to water and food, which was a pelletized AIN-93G diet [30]. Food intake was monitored three times a week. Body weight was measured weekly. Blood glucose was measured every 14 days using a drop of tail blood and a glucometer (FreeStyle Lite–Abbott). Mice were considered diabetic when their blood glucose level was higher than 250 mg/dL. Diabetic animals that lost more than 25% of their body weight were killed before 20 weeks. All procedures were reviewed and approved by the Ethics Committee on Animal Use in Research (CETEA-FMRP, protocol no. 190/2014) in compliance with the Ethical Principles in Animal Research adopted by the National Council for the Control of Animal Experimentation (CONCEA) and following the guidelines for the care and use of laboratory animals [31].

### Sample Size

Sample size was calculated based on the primary outcome in this study, which was immune cell infiltration into pancreatic islets (insulitis). As insulitis is a nominal qualitative variable, the sample size was determined using the following equation:

$$ \frac{\left(\mathrm{p}1.\mathrm{q}1+\mathrm{p}2.\mathrm{q}2\right).{\left( Z\alpha /2+ Z\beta \right)}^2}{{\left(\mathrm{p}1\hbox{-} \mathrm{p}2\right)}^2} $$

Considering that each section of pancreas has approximately 10 islets and that in control groups, approximately 60% have some level of insulitis even in the absence of T1D [32], sample size was established to reach statistical significance when the applied interventions reduced the number of islets with some level of insulitis at least by half (30%).

### Curcumin Supplementation

The AIN-93 diet was prepared twice a week, protected from light, and kept frozen. The food offered to the CUR and TC groups had curcumin added (500 mg/kg). Curcumin was purchased from Sigma-Aldrich (St. Louis, MO, USA). Considering the daily food intake of the mice, the curcumin dose reached approximately 65 mg/kg per day.

### Exercise Training Protocol

All mice from the T and TC groups performed a maximal incremental running test at the beginning of the experimental protocol. This test began at 10 m/min and increased by 1.7 m/min every 2 min until exhaustion (determined when the animal touched the bottom of the bay five times within 1 min). The speed at which exhaustion occurred was considered 100%. Adjustment of exercise load was done every 4 weeks by repeating the same incremental test. According to the animals’ performance in the incremental test, they were divided into three running groups (best, middle, and lower) to guarantee that exercise intensity was maintained and to respect the differences in mouse performance.

Exercise training was carried out for 20 weeks, 5 days a week. All exercise sessions were performed in the morning. The exercise session consisted of a 5-minute warm-up followed by 60 min of running at training intensity and a 2-min cool down. Training intensity varied during week sessions. On Monday, Wednesday, and Friday, the mice ran at 60% of their maximal speed reached in the incremental test; on Tuesday and Thursday, mice had recovery sessions running at 30% of the maximal speed.

### Cytokine and Insulin Concentration

Following overnight fasting, the animals were euthanized by decapitation 48 h after the last exercise session, without previous anesthetic. The serum obtained was used to determine glucose concentration by the colorimetric method (Labtest, Lago Santa, Brazil), and frozen samples (−70 °C) were used to measure insulin, TNF-α, and IL-6 using Luminex xMAP® technology with a Milliplex® kit (Millipore Corporation) analyzed on MAGPIX®; the standard curve of all analytes had *R* ^2^ = 1.

A homeostatic model assessment of insulin resistance (HOMA-IR) was calculated to estimate insulin resistance as previously described [33].

### Insulitis

Pancreas was fixed in formalin (10%), embedded in paraffin, and stained with hematoxylin-eosin. Images were taken on an optical microscope (Olympus BX61VS). Three sections of each pancreas were analyzed. Islets were counted as previously described [34] and labeled as: No Insulitis (absence of immune cells), Pre-Insulitis (few immune cells around the islet), and Insulitis (>25% of cells infiltrated and destruction of islet architecture). Counts were performed manually by two independent researchers (blinded). Insulitis extension was calculated as previously described [35]:

$$ \frac{\left( N \mathit{^{\circ}}\mathrm{No}\ \mathrm{Insulitis}\right)+\left( N \mathit{^{\circ}} \Pr \mathrm{e}\ \mathrm{Insulitis}\times 2\right)+\left( N \mathit{^{\circ}}\mathrm{Insulitis}\times 3\right)}{N \mathit{^{\circ}}\mathrm{Total}\ \mathrm{Islets}} $$

### Statistics

Data are presented as the means ± standard deviation. Analysis of variance (one-way ANOVA) followed by a Tukey post hoc test was performed, except for HOMA-IR and IL-6, which did not have a normal distribution and were analyzed using a Kruskal-Wallis test. Weekly blood glucose was analyzed using a repeated measure ANOVA. A log-rank test was used to compare diabetes incidence. A Chi-square test was used to compare insulitis distribution. The level of significance adopted was *p* < 0.05.

## Results

**Fig. 1:**
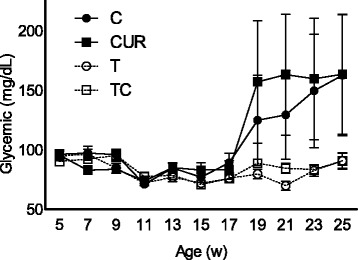
Blood glucose during the 20-week experimental protocol from control (*C*), curcumin (*CUR*), trained (*T*), and trained + curcumin (*TC*). NOD mice were 5 weeks old at the beginning. Data are mean ± standard deviation

Although the daily food intake was approximately 10% greater in the trained group than in the control group, body weight gain was approximately 41% lower in the trained mice after 20 weeks. Only four animals developed T1D, two each in groups C and CUR. Insulin levels tended to be lower in CUR and T, but the difference only reached a statistical level of significance in the TC group. HOMA-IR was not modified by curcumin or exercise training (Table 1). Blood glucose was similar among groups during the 20 weeks, as seen in Fig. 1.

**Table 1:** Food intake, weight gain, T1D incidence, insulin level, and HOMA-IR after the 20-week experimental protocol

Group	Food intake (g/day)	Weight gain (g)	T1D incidence (%)	Insulin (μU/mL)	HOMA-IR
Control	3.05 ± 0.23	12.40 ± 5.02	16.66	28.07 ± 25.53	4.97 ± 4.79
Curcumin	3.13 ± 0.20	9.30 ± 3.30	16.66	15.06 ± 13.15	2.32 ± 1.95
Trained	3.39 ± 0.26^a^	6.73 ± 1.68^a^	0.00	9.60 ± 15.88	1.80 ± 2.95
Trained + curcumin	3.31 ± 0.21	7.25 ± 1.48^a^	0.00	8.27 ± 6.08^a^	1.71 ± 1.17
*P* value	0.0006	0.0006	0.5078	0.0400	0.1172

Data are the mean ± standard deviation

^a^Different from control

**Fig. 2:**
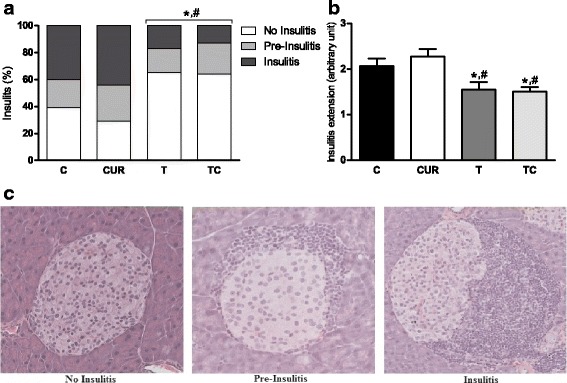
Quantitative/representative analysis of insulitis. Insulitis distribution (**a**), insulitis extension (**b**), and representative image used to label insulitis level (**c**). Control (*C*), curcumin (*CUR*), trained (*T*), and trained + curcumin (*TC*).*Different from control; #different from curcumin

**Fig. 3:**
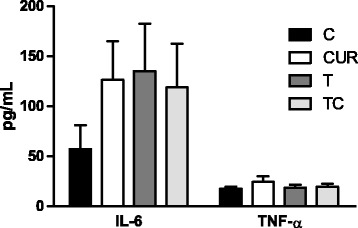
Cytokine levels after the 20-week experimental protocol from control (*C*), curcumin (*CUR*), trained (*T*), trained plus curcumin (*TC*) NOD mice

Despite the low incidence of T1D, insulitis was seen in all groups but in different levels. The trained and trained + curcumin groups had 50% less immune cell infiltration in pancreatic islets than the sedentary groups (Fig. 2a). In addition, insulitis extension was also reduced with exercise training (Fig. 2b). Regarding cytokine levels, IL-6 and TNF-α were not modified by curcumin or exercise training (Fig. 3).

## Discussion

To the best of our knowledge, this study is the first to show that exercise training has the potential to protect pancreatic β cells against an immune response in vivo. However, curcumin supplementation failed to attenuate insulitis in NOD mice.

Curcumin is a polyphenolic compound that exhibits low bioavailability [36]. The route of administration and the dose of curcumin used in this study were established based on previous studies that aimed to simulate a rich polyphenol diet [36–39]. Although chronic oral curcumin (500 ppm in diet) reached detectable concentrations in plasma (0.035 μg/ml) and in brain tissue (0.469 μg/ml) in a dose-dependent study [37], our study indicates that this concentration was not enough to prevent an immune response against pancreatic β cells. However, intraperitoneal curcumin supplementation (25 mg/kg body weight) inhibited leucocyte infiltration in accelerated murine models of T1D [40]. These data suggest that the concentration of curcumin required to modulate immune function could not be reached using dietary strategies.

The low incidence of T1D observed was expected because animals were fed a gluten-free diet and were not in a germ-free environment [41, 42]. However, NOD mice present ~60% immune cell infiltration in islets even in the absence of T1D [32], which is consistent with our findings. Exercise training lowered this rate to 30%.

The effect of exercise training in diminishing body weight gain is well documented and was observed in the present study, showing the importance of physical exercise for caloric balance. Lower body weight gain could be one mechanism that explains the reduction of insulitis in the T and TC groups. Since obesity induces chronic inflammation [43] and the Overload Hypothesis proposes that environment factors, such as obesity, increase T1D risk [12], this line of thought is strengthened.

Another possible mechanism to explain the effects of exercise training is through dendritic cells modulation. It is well known that dendritic cells modulate both innate and adaptive immune responses, and the role of these cells on the development of T1D was recently demonstrated [44]. Several studies have shown that exercise training decreases the number of dendritic cells or diminishes their response [45–48]. Thus, exercise could mitigate autoimmunity by shaping dendritic cell activation.

Regarding cytokine signaling, IL-6 has been recognized as one of the myokines produced during exercise training [49]. In 2010, exercise training was documented as an anti-inflammatory approach, which is able to prevent type 2 diabetes, cardiovascular diseases, cancer, and dementia [50]. Although no significant differences were seen in cytokine levels, the CUR, T, and TC groups exhibited an IL-6 concentration more than twofold higher than the C group (*p* = 0.07). In an ex vivo study, the pancreas of trained animals had fewer apoptosis biomarkers than sedentary animals. When an IL-6 blocker was added to the trained animals’ pancreas, the apoptosis biomarkers rose to sedentary levels. The authors concluded that the benefit of exercise training on pancreatic β cell survival is through the IL-6 pathway [51]. However, the results of the present study cannot reinforce the role of IL-6 in preventing insulitis because the CUR group had the same insulitis markers as the C group, which suggests that the effect of exercise training is due to other mechanisms.

## Conclusions

In conclusion, moderate intensity exercise training has the potential to protect pancreatic β cells against an immune response in NOD mice. The limitation of the present study is a lack of mechanisms that establish a causal effect, as well as the lack of an evaluation of other inflammatory markers, and both pancreatic and blood oxidative stress, epinephrine levels, and the characterization of infiltrating immune cells. Those measurements are an important area of future research. Therefore, additional and prospective studies are needed to uncover the mechanisms that explain the link between exercise training and autoimmunity.

## Abbreviations

AIN: American Institute of Nutrition
ANOVA: Analysis of variance
C: Control
CONCEA: Conselho Nacional de Controle de Exeperimentação Animal
CUR: Curcumin
FMRP: Faculdade de Medicina de Ribeirão Preto
HOMA-IR: Homeostatic model assessment of insulin resistance
IL: Interleukin
NOD: Non-obese diabetic
T: Trained
T1D: Type 1 diabetes
TC: Trained + curcumin
TNF: Tumor necrosis factor
